# Role of Epidural Injections to Prevent Surgical Intervention in Patients with Chronic Sciatica: A Systematic Review and Meta-Analysis

**DOI:** 10.7759/cureus.723

**Published:** 2016-08-04

**Authors:** Adnan Bashir Bhatti, Sunny Kim

**Affiliations:** 1 Medical Director of Clinical Research, Spine Surgery, Tristate Brain and Spine Institute; 2 Spine Surgery, Tristate Brain and Spine Institute

**Keywords:** sciatica, chronic back pain, epidural injection, disc herniation, lumbar radiculopathy

## Abstract

Objective: The aim of this study is to evaluate the efficacy of the different types of epidural injections (EI) to prevent surgical intervention in patients suffering from chronic sciatica due to lumbar disc herniation (LDH).

Material and Methods: Studies were identified by searching PubMed, MEDLINE, and Google Scholar to retrieve all available relevant articles. Lists of references of several systematic reviews were also used for scanning further references. Publications from the past ten years (2006-2016) were considered, and all studies selected were in the English language only. The studies employed specified the use of EI to treat sciatica caused by LDH. A total of 19 papers meeting the eligibility criteria (mentioned below) were included in this study. The pain scores, functional disability scores, and surgical rates from these studies were considered, and meta-analysis was performed.

Outcome measures: Pain scores, functional disability scores, and surgical rates were assessed from the included studies. The Numeric Rating Scale (NRS) and Visual Analogue Scale (VAS) have been the most commonly used baseline scales for pain evaluation followed by the Verbal Numerical Rating Scale (VNRS) and Japanese Orthopedic Association (JOA). The Oswestry Disability Index (ODI) and Roland Morris Disability Questionnaire (RMDQ) scales were used for the functional disability scoring system in the literature.

Results: Significant improvement in the pain scores and functional disability scores were observed. Additionally, greater than 80% of the patients suffering from chronic sciatica caused by LDH could successfully prevent surgical intervention after EI treatment with or without steroids.

Conclusion: The management of sciatica with EI treatment results in significant improvements in the pain score, functional disability score, and surgical rate. We concluded that EI provides new hope to prevent surgical intervention in patients suffering from sciatica caused by LDH.

## Introduction

Medical literature refers to sciatica as a lumbosacral radicular syndrome, lumbar radiculopathy, nerve root pain, and nerve root entrapment/irritation. It is characterized by pain radiating from the back into the leg [1-3]. It is a common and debilitating symptom rather than a specific diagnosis. It may be caused by lumbar disc herniation (LDH), lumbar canal or foraminal stenosis, and or inflammatory processes around the nerve root [4]. Among all the causes, LDH is the most common cause of sciatica leading to surgical interventions [5] and was first reported by Mixter and Barr in 1934. According to some estimates, the prevalence of sciatica caused by LDH approaches 9.8 per 1,000 cases, of which 3.7% are in women and 5.1% in men [6]. In the general population, the sciatica is reported in 1-2% of these cases; LDH has been reported to occur in 90% [7].

There is a general agreement that sciatica due to LDH is frequently a self-limited condition, and therefore, most of these patients will improve within weeks to months without any medical intervention. A few patients may require conservative management such as rest, analgesics, traction, medication, physical therapy, or a structured exercise program. Nevertheless, for the patients who are refractory to four to six months of conservative management or their pain is progressive under the conservative management, in such patients surgical intervention is recommended [8]. The primary aim of any surgical intervention is to provide rapid relief from pain and functional disability [5]. Surgical intervention is rapidly effective, but it is a costly procedure and associated with several post-operative complications, including but not limited to chronic pain and persistent disability [9]. Moreover, long-term outcomes of conservative management have been reported to be better than surgical intervention in several studies [10-11]. Additionally, surgical intervention is not available for everyone who is symptomatic and may lead to failure in approximately 25% of carefully selected cases [12].

Sciatica occurs most commonly due to herniation of a lumbar intervertebral disc, resulting in an inflammatory response around the nerve root. This inflammatory response rather than mechanical compression is the primary cause of the radicular pain. Therefore, anti-inflammatory drugs are used to reduce pain by reducing the inflammation around the nerve [2]. Several minimally invasive (MI) anti-inflammatory treatments such as segmental epidural steroid injections (ESI), selective nerve root blocks, disc decompression using laser energy (laser discectomy), radiofrequency coblation (nucleoplasty), intradiscal oxygen-ozone (O2-O3) injection for treating disc-related radiculopathy as an alternatives to surgical intervention [9] have been devised.

Among various modalities applied in the management of painful conditions of the spine, EI is one of the most commonly utilized non-surgical interventions [13]. All the procedures mentioned above are either associated with side effects or lesser effectiveness [9]. Thus, EI seems to be the best non-surgical alternative treatment option available for severe cases of sciatica. The use of EI for the management of lower back pain and sciatica was initiated in 1900 in Paris by Jean Sicard and Fernand Cathelin [14]. At present, EIs are administered in the lumbar spine by three different approaches namely caudal, interlaminar, and transforaminal [13]. Different types of steroids have been used in these injections including triamcinolone, methylprednisolone, betamethasone, and dexamethasone [14]. These EIs are aimed at providing analgesia for a variable duration during which the patient can go for rehabilitation exercises [15]. It has been hypothesized that EI is a better alternative treatment option than surgical intervention for patients who do not wish to undergo more invasive procedures. It is evidenced by the fact that many chronic back pain patients visit the pain management clinics every couple of weeks to get repeated epidural injections [14].

Various systematic reviews over time evaluated the efficacy of EI by comparing its outcomes with conservative management. Nevertheless, to the best of our knowledge, there has been no systematic review and meta-analysis of the potential role of EI in preventing surgical intervention. This study assessed the possible role of EI in preventing surgical intervention based on the outcome measures assessment after EI treatment in the past ten years from 2006 to 2016.

## Materials and methods

In this study, we aimed to update the literature on the potential role of EI in preventing surgical intervention based on outcome measures assessment after treatment with EI for sciatica or radiculitis caused by LDH. To accomplish this purpose, we searched databases like MEDLINE, PubMed Central, Google Scholar, and included papers published between 2006 to 2016. We reviewed literature in the English language only. Lists of references of several systematic reviews were also used for scanning further references.

### Data extraction

Combinations of the following keywords were used for data extraction: lumbar disc herniation, lumbosciatic pain, radicular pain, radiculitis, sciatica, epidural injection, epidural steroid, epidural perineural injection, interlaminar epidural, intra-articular corticosteroid, transforaminal injection, caudal epidural injection.

**Figure 1:**
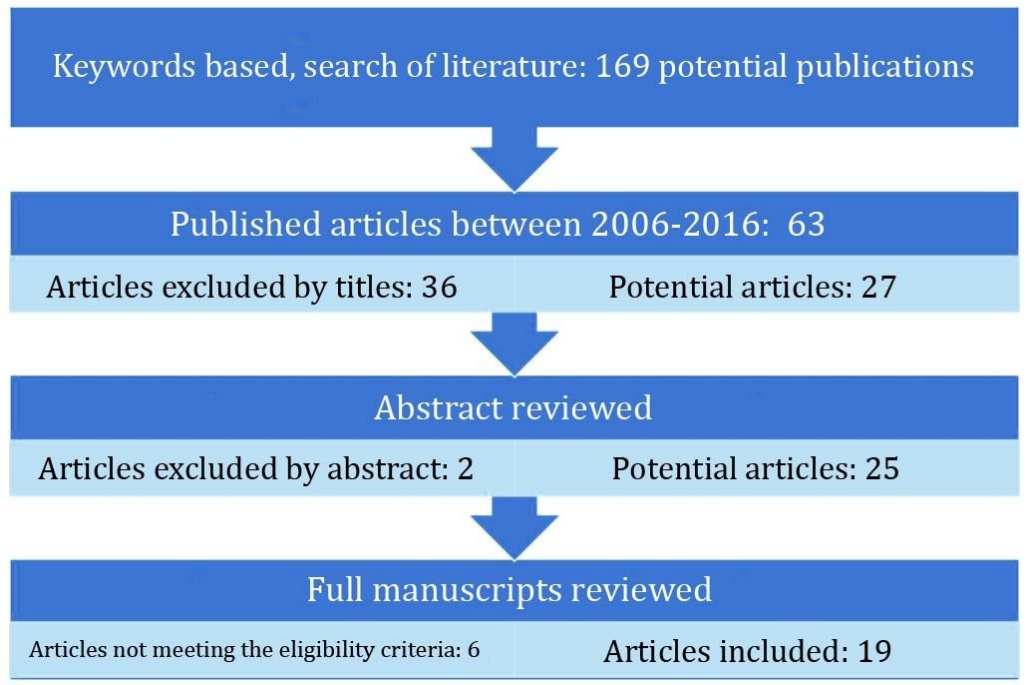
Flow Chart of the Included Studies.

The result was the identification of 169 relevant papers, 63 of which were found to be published in the past 10 years. Scanning titles, we found 27 of the 63 papers relevant to our study based on the selection criteria mentioned later in the text. A lack of included information led to the exclusion of 2 papers. Further, 6 more papers were excluded due to the absence of surgical rate data, or sciatica was associated with the causes other than LDH. Figure 1 summarizes the results of the literature search and inclusion steps of the studies.

### Selection criteria

Types of Studies 

Randomized controlled trials (RCT), prospective observational studies, retrospective studies, double-blind clinical trials, and full-text journal articles published in peer-reviewed journals were included in this study. Table 1 summarizes the details of the included studies.

**Table 1:** Literature Map TFEI = Transforaminal epidural injections; ILEI = Interlaminar lumbar epidural injections; CEI = Caudal epidural injections; LEI = Lumbar epidural injections; NA = Not available; FU = Follow-up.

Study	Study Design	Injection	Mean Age	Sample Size	Gender (%)		Diagnosis	Pain Duration (Weeks)	Maximum FU (Months)
					Females	Males			
Sayegh (2009) [25]	Prospective, randomized, double-blind clinical trial	CEI without steroid	49.12	183	32.78%	67.23%	Lumbar radicular pain and sciatica	>4	12
Ghahreman (2010) [3]	Prospective, randomized study	TFEI with steroid	49.5	150	40.66%	59.30%	Lumbar disc herniation	12	12
Manchikanti (2014 A) [17]	Randomized, controlled, double-blind, active control trial	TFEI with or without steroids	42.85	120	50%	50%	Lumbar disc herniation and radiculitis	24	24
Manchikanti (2014 B) [12]	Randomized, controlled, double-blind, active control trial.	LIEI with or without steroids	44.5	120	69.20%	30.80%	Lumbar disc herniation and radiculitis	24	24
Manchikanti (2011) [21]	Randomized, controlled, double-blind trial	CEI without steroid	45.85	120	65%	35%	Lumbar disc herniation and radiculitis	24	12
Manson (2013) [18]	Retrospective	TFEI with steroids	45.8	91	41.75%	58.24%	Lumbar disc herniation and/or radiculopathy	6	26.6
Manchikanti (2008) [26]	A randomized, double-blind, equivalence trial.	CEI without steroid	47.05	84	66.60%	33.33%	Lumbar disc herniation and radiculitis	24	12
Owlia (2007) [28]	Comparative study	LEI with steroid	38.2	84	57.14%	42.80%	Lumbar radicular pain	>2	3
Kennedy (2014) [1]	Multicenter, double-blind, prospective, randomized trial	TFEI with steroids	35.75	78	34.61%	65.38%	Unilateral radicular pain	8	6
Manchikanti (2010) [23]	Randomized, double-blind, controlled trial	LIEI with injections	41.95	70	65.71%	34.26%	Lumbar radicular pain	24	12
Helvoirt (2014) [16]	Prospective cohort study	TFEI with steroids	47.3	69	50.70%	49.30%	Lumbar disc herniation	20	12
Rados (2011) [22]	Randomized prospective study	TFEI & LIEI with steroid	49.2	64	35.90%	64.10%	Chronic lumbar radicular pain caused by herniated disc	12	6
Spijker-Huiges (2014) [2]	Randomized controlled trial	TFEI with steroids	43.7	63	47.61%	52.38%	Lumbosacral radicular syndrome	2-4	13
Gomez (2007) [27]	Retrospective	LIEI with steroid	47	60	50%	50%	Lumbosciatic pain	24-48	6
Baral (2011) [20]	Prospective observational study	TFEI with steroid	41.04	50	48%	52%	Lumbar disc herniation	NA	6
Kawu (2012) [19]	Prospective-controlled observational study	TFEI & LIEI with steroids	47.6	49	NA	NA	Lumbar disc herniation	31.2	6
Schaufele (2006) [29]	Retrospective	LIEI Versus TFEI without steroid	NA	40	NA	NA	Lumbar intravertebral disc herniation	2-48	12
Laiq (2009) [24]	Quasi-experimental study	LEI with steroid	40.5	25	68%	32%	Lumbar radicular pain	2	6
Yang (2006) [30]	Prospective	TFEI with steroid	50	19	42.10%	57.89%	Sciatica with unilateral symptoms	8-96	24

Types of Participants

Patients who had sciatica with the pain duration of more than 2 weeks secondary to LDH were included in the study.

Types of Epidural Injection Approaches

Three different approaches have been used in the literature: lumbar transforaminal epidural injection (TEI), lumbar interlaminar epidural injection (IEI), and caudal epidural injections (CEI).

Types of Outcome Measures

All possible available outcome measures such as pain scores, functional disability scores, and surgical rates were assessed from the literature, and meta-analysis was performed. The Numeric Rating Scale (NRS) and Visual Analogue Scale (VAS) were observed to be the most commonly used scales for pain evaluations followed by the Verbal Numerical Rating Scale (VNRS) and Japanese Orthopedic Association (JOA). The Oswestry Disability Index (ODI), Roland Morris Disability Questionnaire (RMDQ), and International Classification of Impairments, Disabilities, and Handicaps (ICIDH) by WHO Grade score were considered for the functional disability scoring system in the literature.

## Results

The average patient sample size was 81 in the included studies. Females were slightly more affected with sciatica secondary to LDH than males, with an average percentage of 51% and 49%, respectively. All patients were adults with a relatively young age; the mean age was 45 ±4 years. Follow-up time reported in the studies ranged from three months to two years.

### Pain score

Pain score has been assessed in 16 studies [1-2, 12, 16-17, 19-24, 26-30]. The NRS was used in 7 studies [1-2, 12, 17, 21, 23, 26], VAS used in 7 studies [16, 19-20, 22, 24, 27-28], and VNRS and JOA used in the remaining 2 studies [29-30].

In those studies where pain assessment was documented using the NRS scale, the mean baseline scores pre-treatment in two studies [2, 12] were observed to be 7.7-8.25, which reduced post-injection to 1.3-4.1, respectively, at the last follow-up; that is a 50-83% improvement from the pre-injection state, as shown in Table 2.

**Table 2:** Pain Scores NRS = Numeric Rating Scale; VAS = Visual Analogue Scale; VNRS = Verbal Numerical Rating Scale; JOA = Japanese Orthopedic Association (JOA).

Study	Baseline	Last Follow-up	% Improvement
Studies reported NRS			
Spijker-Huiges (2014) [2]	7.7	1.3	83
Kennedy (2014) [1]	6.9	1.31	81
Manchikanti (2010) [23]	8	3.6	55
Manchikanti (2008) [26]	7.95	3.6	55
Manchikanti (2011) [21]	7.95	3.8	52
Manchikanti (2014 A) [17]	8.1	3.9	52
Manchikanti (2014 B) [12]	8.25	4.1	50
Studies reported VAS score			
Helvoirt (2014) [16]	52.3	8.99	83
Kawu (2012) [19]	77.6	39.6	49
Baral (2011) [20]	6.98	3.68	47
Rados (2011) [22]	7.04	3.9	45
Laiq (2009) [24]	6	6	0
Gomez (2007) [27]	VAS 10 (very severe pain) Patients (30%)	VAS 10 (very severe pain) Patients (6.7%)	NA
	VAS 6-9 (severe pain) Patients (50%%)	VAS 6-9 (severe pain) Patients (21.7%%)	
	VAS 3-5 (moderate pain) Patients (20%)	VAS 3-5 (moderate pain) Patients (11.7%)	
	VAS 1-2 (mild pain) Patients (0%)	VAS 1-2 (mild pain) Patients (25%)	
	VAS 0 (no pain) Patients (0%)	VAS 0 (no pain) Patients (35%)	
Owlia (2007) [28]	NA	NA	58
Studies reported VNRS			
Scahufele (2006) [29]	6.6	4.55	31
Study reported JOA			
Yang (2006) [30]	14.26	23.38	

Further, among 7 studies reporting the VAS scale, the study by Helvoirt et al., (2014) [16] mean baseline value was 52.3 that reduced to mean value of 8.99 at the last follow-up, which is an 83% improvement in the pain score. In another study of Kawu et al., (2012) [19] mean baseline value of 77.6 pre-treatment was reduced to 39.6, approximately a 50% improvement. Further, 3 studies reported mean baseline VAS scores were greater than 6 [20, 22, 24]; among these, in Baral et al., [20] the value reduced to 3.68, and in Rados et al., (2011) [22] the value reduced to 3.9. However, in the remaining 1 study by Laiq et al., (2009) [24], no statistically significant improvement was observed, but 68% of the patients reported pain relief. In another study by Gomez et al., [27] baseline VAS score was ≥6 in 80% patients, and none of the patients had a VAS score of 0-2 at the baseline. At the last follow-up, greater than 70% of the patients achieved a VAS score between 0-5 with 60% of the patients having a VAS score of 0-2, representing a significant improvement post-EI treatment. Owlia et al., (2007) [28] also reported improvement of 65.4%, 75%, and 58.3% at the follow-up of two weeks, one month, and three months, respectively.

Further, in 2 studies [29-30] reporting VNRS and JOA scores for pain evaluation, a statistically significant improvement was observed. In 1 study reporting a VNRS score, a 31% improvement was observed [29]. In the other study by Yang et al., (2006) [30] significant improvement of the JOA score from 14.26 at baseline to 23.38 at the last follow-up was reported. Thus, the literature published in the past ten years has reported a significant improvement in the pain scores, and no study was found to be reporting a worsening of the pain score. The details about the pain scores are given in Table 2.

### Functional disability score

There was a total of 13 studies in which four different types of functional disability scoring systems were reported. The ODI scores are reported in 9 studies [12, 17, 19-23, 25-26], RMDQ in 2 studies [2, 16], International Classification of Impairments, Disabilities, and Handicaps (ICIDH) by WHO Grade score in 1 study [27], and JOA score in 1 study [30]. In all of these studies, a significant improvement after EI was observed.

Among 9 studies reporting ODI scores for functional disability, 5 considered ≥50% reduction in ODI as a significant clinical improvement [1, 12, 17, 21, 23], 1 considered ≥40% reduction as a significant clinical improvement [26], and 1 considered >10 points or ≥20% change as a significant clinical improvement [20].

Kennedy et al., (2014) [1] reported a baseline ODI measure of "severe disability" at the score range 40-60, which reduced to "minimal disability" at the range of 0-20 at the last follow-up. In 5 studies by Manchikanti et al., [12, 17, 21, 23, 26] the ODI mean baseline value of 29 reduced to 14 at the last follow-up, indicating a >50% improvement in the functional disability score. Further, greater than 75% improvement was reported in two groups by Sayegh et al (2009) [25]. In two studies by Kawu et al., (2012) [19] and Baral et al., (2011) [20] the mean baseline ODI of >60 was reported, which moved to 32 and 35.68, respectively, at the last follow-up, which is greater than a 40% improvement. Rados et al., [22] reported only a 26% improvement post-EI.

Further, among 2 studies [2, 16] reporting the RMDQ scoring system, the mean baseline scores of 12.2-16.5 moved significantly to 3.3-2.3 at the last follow-up, which indicates an 80% improvement.

A study by Gomez et al., (2007) [27] reported a grading score system by WHO; Grades 2 & 3 and Grade 1 had 65% and 35% of patients, respectively; no patient was in Grade 0. Post-injection average of 76% of the patients moved to Grade 0-1 at the last follow-up, and 50% were in Grade 0. The study by Yang et al., (2006) [30] also reported significant improvement in the JOA score of daily activity at the last follow-up.

Thus, in the literature published in the past ten years, significant improvement in functional disability scores post-injection has been observed. The details about the functional disability scores are given in Table 3.

**Table 3:** Functional Disability Scores ODI = Oswestry Disability Index; RMDQ = Roland Morris Disability Questionnaire; ICIDH = International Classification of Impairments, Disabilities, and Handicaps; JOA = Japanese Orthopedic Association.

Study	Mean Baseline	Follow-up	% Improvement
Studies reported ODI			
Baral (2011) [20]	60.86	35.68	41
Kawu (2012) [19]	61	32	48
Rados (2011) [22]	~54	~40	26
Sayegh (2009) [25]	38.5	8.95	77
Manchikanti (2014 A) [17]	29.95	14.8	51
Manchikanti (2014 B) [12]	28.95	14.5	50
Manchikanti (2011) [21]	28.55	14.3	50
Manchikanti (2010) [23]	29.35	14	52
Manchikanti (2008) [26]	28.55	13.3	53
Studies reported RMDQ			
Helvoirt (2014) [16]	12.2	3.3	73
Spijker-Huiges (2014) [2]	16.5	2.3	86
Studies reported ICIDH by WHO			
Gomez (2007) [27]	Grade 3 (11.7%)	Grade 3 3.3%	NA
	Grade 2 (53.3%)	Grade 2 20%	
	Grade 1 (35%)	Grade 1 26.7	
	Grade 0 (0%)	Grade 0 50%	
Studies reported JOA			
Yang (2006) [30]	7.44 ±2.16	12.19 ± 2.23	

### Surgical rate

Among 19 studies, the surgical rate has been reported in 9 studies [1, 16, 18-20, 24-25, 29-30]. Based on the literature review of past ten years, we found that surgical rate post-EI treatment was between 13% and 21% in 7 studies [1, 16, 20, 24-25, 29-30], depicting that greater than 78% of the patients were able to prevent surgical intervention. Additionally, in 1 study [19], the surgical rate was found to be only 7.05% post-EI treatment, which showed a greater than 90% reduction in surgical rate. The highest surgical rate was 44% in one study [18], and thus only 56% of the patients could prevent surgical intervention. Overall, on an average of 80%, patients were able to avoid surgical intervention with the help of EI treatment. The details about the surgical rates are given in Table 4.

**Table 4:** Surgical Rate After Epidural Injection Treatment EI = Epidural injection.

Study	Average Surgical Rate After EI	Average Non-Surgical Rate After EI
Kawu (2012) [19]	7.05%	92.95%
Baral (2011) [20]	13.00%	87.00%
Yang (2006) [30]	15.80%	84.20%
Kennedy (2014) [1]	16.75%	83.25%
Schaufele (2006) [29]	17.50%	82.50%
Sayegh (2009) [25]	17.50%	82.50%
Laiq (2009) [24]	20.00%	80.00%
Helvoirt (2014) [16]	21.70%	78.30%
Manson (2013) [18]	44.00%	56.00%

## Discussion

We found three different approaches of EI: transforaminal, interlaminar, and caudal. These have been used in the management of sciatica to prevent surgical intervention. Each approach has its own advantage and utility. For instance, the caudal approach has the advantage of avoiding dural puncture, and it can be used in cases where previous surgeries have been done. The advantage of the interlaminar approach includes the ability to treat both unilateral as well as bilateral pain, and it is highly likely that the injected medication will reach adjacent spinal levels [31]. In this review among the 19 studies, the transforaminal approach was observed to be used in 11 studies [1-3, 16-20, 22, 29-30], and thus found to be the most common approach. This may be due to the associated advantage of giving diagnostic information that may indicate the cause of sciatica by blocking the specific root rather than affecting the entire thecal sac [30]. It has been observed that the transforaminal approach is target-specific and reported as the best route to deliver medication to the ventral epidural space and dorsal root ganglion [31]. Additionally, among different approaches, the transforaminal approach requires the smallest volume to reach the primary site of pathology [24].

Further, the transforaminal approach with steroids (triamcinolone, methylprednisolone, betamethasone, and dexamethasone) is found to be a common treatment option for patients with low back pain or sciatica [14, 32]. We observed that particulate steroids [1-2, 17-18, 22-25, 27-28, 30] such as triamcinolone, methylprednisolone, and betamethasone have been used more commonly in the past ten years than the non-particulate steroids like dexamethasone. However, non-particulate steroids, such as dexamethasone have been suggested as a better option in the literature as they can avoid the potential complication of embolization of particulate steroids, such as Depo-Medrol into the feeder vessels of the spinal cord causing paraplegia [33].

### Outcome measures

The outcome measures assessed in this study are the pain scores, functional disability scores, and the surgical rates after treatment with EI. We assessed whether EIs are capable of preventing surgical intervention or not. We included the studies in which the patients experienced pain for at least two weeks in duration without any relief with the conservative management. Most of these patients were referred for surgical intervention. However, in order to avoid surgical intervention and associated psychological depressive effects, they underwent EI treatment.

A total of 19 studies included in this study, pain scores (Table 2), functional disability scores (Table 3), and subsequent evaluation data were given in 16 and 13 studies, respectively. Both of the outcome measures show significant improvements post-injection.

Surgical rates in the past ten years due to LDH has been reported in 9 studies [1, 16, 18-20, 24-25, 29-30], which show a significant reduction in surgical intervention after treatment with EI.

Based on the outcome measures assessment, we have come to the conclusion that an average of greater than 80% of the patients was successful in preventing surgical intervention after the treatment with EI, which proves our point of preventing surgical intervention with the help of EI treatment in patients suffering from sciatica caused by LDH.

### Limitations and future recommendations

In a recent systematic review by Pountos (2015) et al., [33], several complications such as stroke, damage to the neural element, and death with EI have been reported, but these complications were mostly anecdotal, and actual incidence is unknown. It seems that some individuals may have a high risk of developing complications after receiving EI. Therefore, more research is required to identify those high-risk individuals. In this systematic review, we have not assessed either the short-term or long-term complications of EI with or without steroids. It is due to the fact that most studies failed to report these evaluations. Thus, these evaluations of EIs for the management of sciatica can be done in future studies.

## Conclusions

This study reveals that appropriate use of EI to treat sciatica could significantly improve the pain score and functional disability score, which leads to significant decrease in surgical rate. Additionally, EIs with or without steroids are clinically effective, fast, safe, and a less expensive treatment method as compared to surgical intervention. We concluded that treatment with EI significantly reduces the rate of surgical intervention in patients suffering from chronic sciatica caused by LDH.
